# Supplementary material for: Microfluidic-Assisted Fabrication of Monodisperse Core–Shell Microcapsules for Pressure-Sensitive Adhesive with Enhanced Performance
Source: Nanomaterials (Basel). 2020 Feb 6;10(2):274. doi: 10.3390/nano10020274 (PMC7075162; doi:10.3390/nano10020274)
Supplement: Supplementary file 1 [file nanomaterials-10-00274-s001.pdf]

Supplementary Materials

Microfluidic-Assisted Fabrication of Monodisperse Core-Shell Microcapsules for Pressure-Sensitive Adhesive with Enhanced Performance

Xiangshen You [1,#], Bingsheng Wang [1,#], Shuting Xie [1], Lanhui Li [1], Han Lu [1], Mingliang Jin [1,2], Xin Wang [1,2], Guofu Zhou [1,2] and Lingling Shui [1,2,3,*]

[1] Guangdong Provincial Key Laboratory of Optical Information Materials and Technology, South China Academy of Advanced Optoelectronics, South China Normal University, Guangzhou 510006, Guangdong, China; xiangshenyou@m.scnu.edu.cn (X. Y.); bestbs@m.scnu.edu.cn (B.W.); stxie@m.scnu.edu.cn (S.X.) lanhui.li@m.scnu.edu.cn (L.L.); hanlu@m.scnu.edu.cn (H.L.); jinml@scnu.edu.cn (M.J.); wangxin@scnu.edu.cn (X.W.); guofu.zhou@m.scnu.edu.cn (G.Z.)

[2] International Academy of Optoelectronics at Zhaoqing, South China Normal University, Guangzhou 510631, Guangdong, China;

[3] School of Information and Optoelectronic Science and Engineering, South China Normal University, Guangzhou 510006, Guangdong, China

* Correspondence: shuill@m.scnu.edu.cn; Tel.: +86-20-3931-0508

[#] These two authors contributed equally to this work

Table S1. Composition and properties of the fluidic phases used in this work.

Fluidic phase	Component	Density (g/cm³)	Viscosity (mPa·s)	Interfacial tension (mN/m)
Inner	Ethylene glycol solution containing 30 wt.% oxalic acid & 0.05 wt.% methyl orange	1.2141	-	3.87
Middle	ETPTA with 0.1 wt.% 2-dimethoxy-2-phenylacetophenone	1.0851	64.8742	-
Outer	Glycerol aqueous solution containing 4 wt.% PVA	1.0748	1.9154	18.81

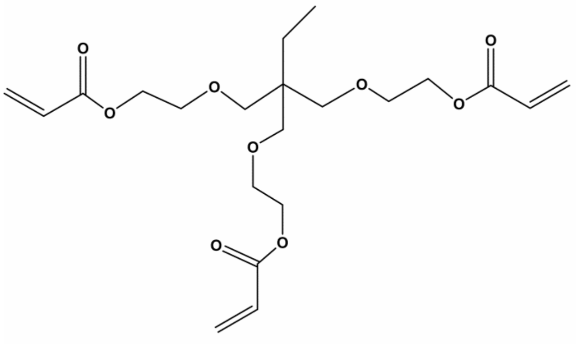

Scheme S1. Monomer structure of ETPTA used for the capsule preparation.

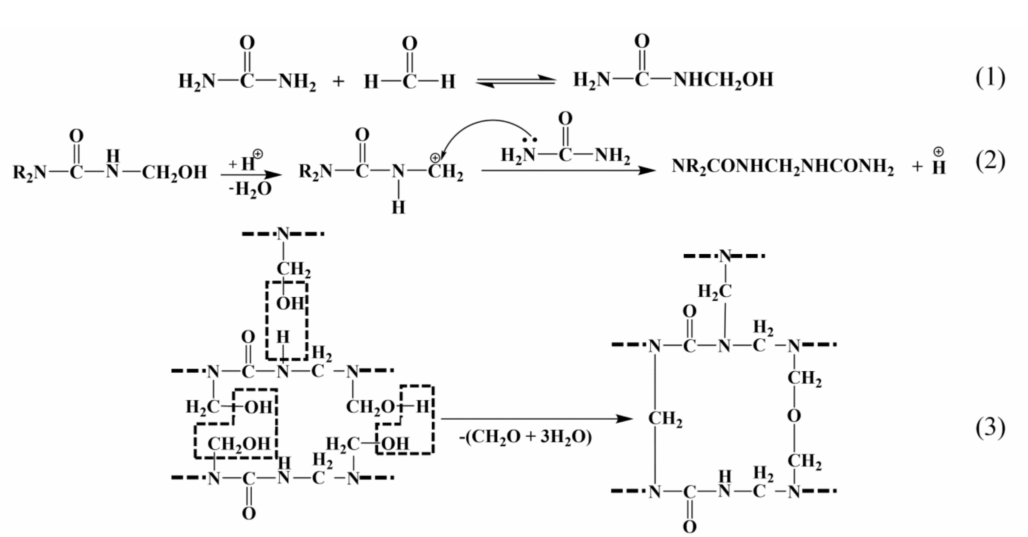

Scheme S2. Urea-formaldehyde resin reaction mechanism. (1) UF resin prepolymer prepared by basic methylolation. (2) Chain growth reaction under acidic condition. (3) Formation of macromolecular structure of urea-formaldehyde resin after curing.

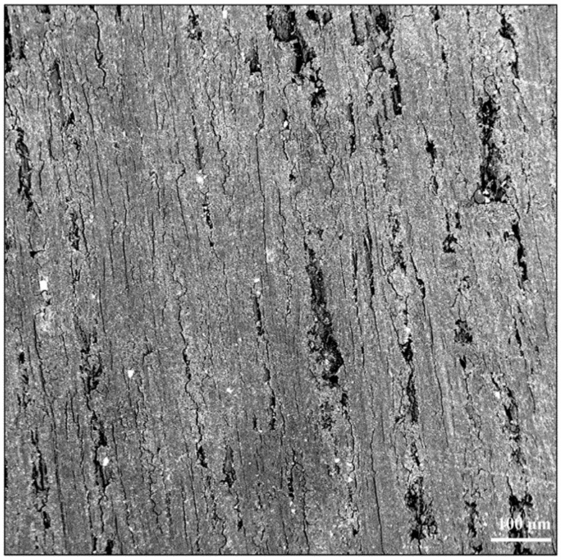

Figure S1. SEM image of plywood surface.

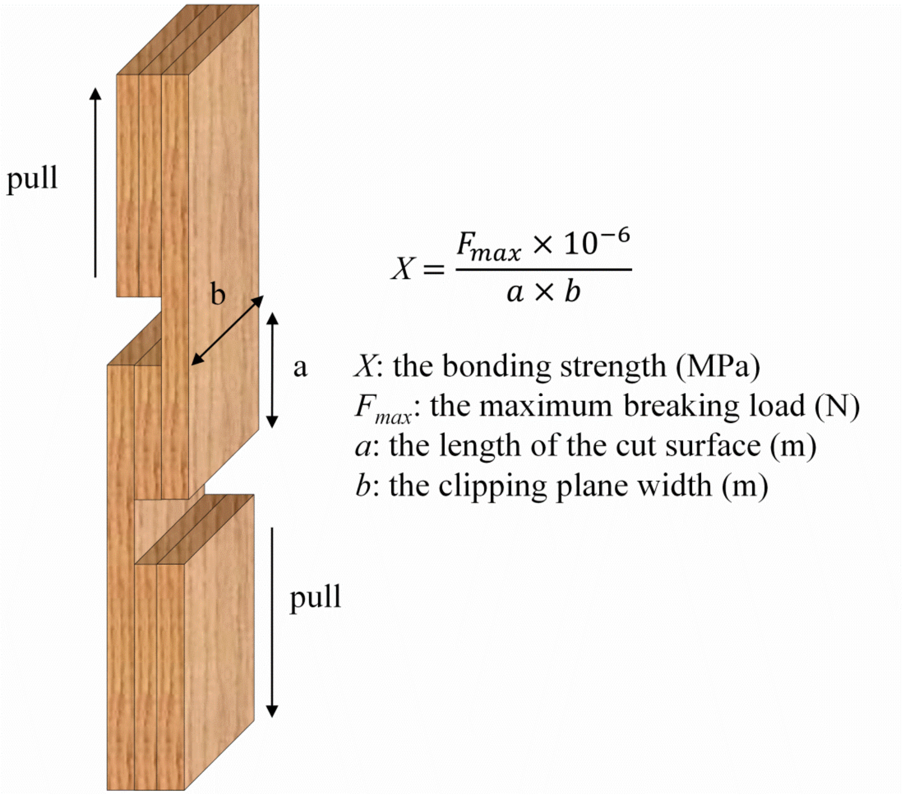

Figure S2. Schematic drawing of the bonding strength measurement
